# The impact of multimorbidity patterns on health-related quality of life in the general population: results of the Belgian Health Interview Survey

**DOI:** 10.1007/s11136-021-02951-w

**Published:** 2021-08-23

**Authors:** Lisa Van Wilder, Brecht Devleesschauwer, Els Clays, Stefanie De Buyser, Johan Van der Heyden, Rana Charafeddine, Pauline Boeckxstaens, Dirk De Bacquer, Sophie Vandepitte, Delphine De Smedt

**Affiliations:** 1 Department of Public Health and Primary Care, Ghent University, University Hospital, Corneel Heymanslaan 10 4K3, 9000 Ghent, Belgium; 2 Department of Epidemiology and Public Health, Sciensano, Brussels, Belgium; 3 Department of Veterinary Public Health and Food Safety, Ghent University, Merelbeke, Belgium; 4 Biostatistics Unit, Department of Public Health and Primary Care, Ghent University, Ghent, Belgium

**Keywords:** Health-related quality of life, Multimorbidity, Chronic disease, EQ-5D

## Abstract

**Background:**

Chronic diseases and multimorbidity are a major cause of disease burden—for patients, caregivers, and society. Little is known however about potential interaction effects between specific disease combinations. Besides an additive effect, the presence of multiple conditions could also act synergistically or antagonistically regarding the impact on patients’ health-related quality of life (HRQoL). The aim was to estimate the impact of coexisting chronic diseases on HRQoL of the adult general Belgian population.

**Methods:**

The Belgian Health Interview Survey 2018 provided data on self-reported chronic conditions and HRQoL (EQ-5D-5L) for a nationally representative sample. Linear mixed models were used to analyze two-way and three-way interactions of disease combinations on HRQoL.

**Results:**

Multimorbidity had a prevalence of 46.7% (≥ 2 conditions) and 29.7% (≥ 3 conditions). HRQoL decreased considerably with the presence of multiple chronic diseases. 14 out of 41 dyad combinations and 5 out of 13 triad combinations showed significant interactions, with a dominant presence of negative/synergistic effects. Positive/antagonistic effects were found in more subjective chronic diseases such as depression and chronic fatigue. Conditions appearing the most frequently in significant disease pair interactions were dorsopathies, respiratory diseases, and arthropathies.

**Conclusions:**

Diverse multimorbidity patterns, both dyads and triads, were synergistically or antagonistically associated with lower HRQoL. Tackling the burden of multimorbidity is needed, especially because most disease combinations affect each other synergistically, resulting in a greater reduction in HRQoL. Further knowledge about those multimorbidity patterns with a greater impact on HRQoL is needed to better understand disease burden beyond mortality and morbidity data.

**Supplementary Information:**

The online version contains supplementary material available at 10.1007/s11136-021-02951-w.

## Introduction

Chronic diseases are highly prevalent and responsible for 73% of all deaths globally [1, 2]. Moreover, about half of the patients with a chronic disease suffer from multimorbidity, defined as the co-occurrence of multiple chronic conditions in a given individual [3]. Chronic diseases and in particular multimorbidity are a major cause of disease burden—for patients, caregivers, and society at large [4, 5]. Mortality measures alone are inadequate to capture the full impact of chronic diseases [6]. Hence, the interest into patients’ health-related quality of life (HRQoL) measures is emerging. HRQoL captures individuals’ self-perceived impact of a medical condition, its symptoms, and treatment referring to physical, mental, and social well-being, compared to what they believe to be ideal [5, 7]. Evidence shows a disease-associated loss in HRQoL, with HRQoL being an important predictor of morbidity and mortality [8, 9].

HRQoL loss in single chronic diseases has already been explored in several studies, however, the impact of combinations of diseases is understudied [1, 10–13]. Little is known about the interaction effect between different diseases on HRQoL. Besides an additive effect, multiple conditions could also act synergistically or antagonistically to impact HRQoL [14]. Previous research suggests synergetic effects between combinations of physical and mental conditions, however, in depth knowledge about the effect of specific pairs is lacking [15–17]. Given that multimorbidity affects an increasing number of people, knowledge on how this impacts self-perceived health is of major importance [18, 19].

The Belgian Health Interview Survey (BHIS) collected data on chronic diseases and HRQoL in a large representative sample of the general Belgian population [20, 21]. These data allow for gaining a better understanding of potential interaction effects between specific chronic diseases, which could be of major interest to researchers, clinicians, and policy makers worldwide. This study aims to estimate how the co-occurrence of different chronic diseases can impact a person’s HRQoL.

## Methods

### Belgian Health Interview Survey

Data from the BHIS 2018 were used [22]. BHIS is a cross-sectional household survey that includes a representative sample of the Belgian population through multistage stratified sampling, involving a geographical stratification, a selection of clusters within each stratum, a selection of households within each cluster, and a selection of individuals within each household. In 2018, 11,611 individuals were interviewed with a response rate of 57% at the household level. Data collection were undertaken using face-to-face interviews (to obtain socio-demographic information) and supplemented with a self-administered questionnaire (to obtain HRQoL data). Details on methodology of the BHIS can be found elsewhere [23].

### Measures

Prevalence of chronic diseases was self-reported, based on the following question: ‘Have you had one of the following disease or condition in the past 12 months?’. Participants indicated on a list of 38 diseases whether they had suffered from a certain disease with the responses ‘yes’ or ‘no’. The list also included chronic conditions (e.g. hypertension), consequences of chronic disease (e.g. hip fracture), and acute diseases with chronic consequences (e.g. stroke). The 38 chronic conditions were mapped into 23 chronic diseases or disease groups because many conditions are affecting the same body system. The mapping was based on the ICD-10 and a multimorbidity questionnaire (Table 1) [24, 25].

**Table 1:**
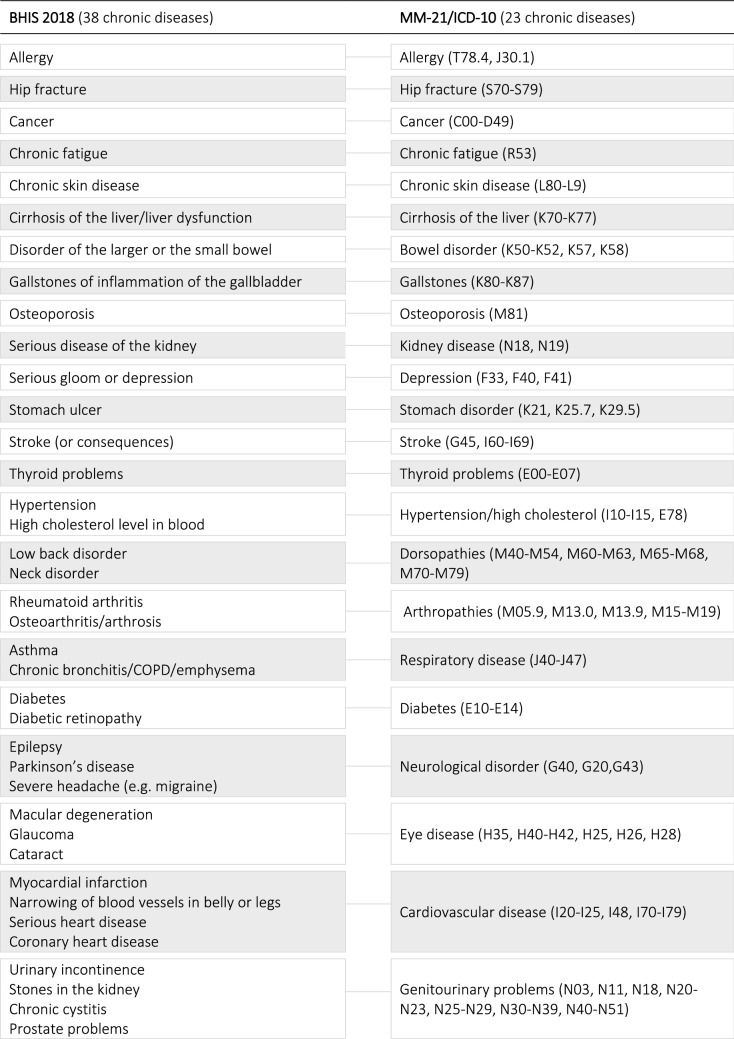
Mapping of the chronic diseases according to the MM-21 and ICD-10

To date, consensus on the definition of multimorbidity is still lacking. The definition of ≥ 2 chronic diseases is under discussion, especially when highly prevalent conditions (e.g. hypertension) are included because these may result in high prevalence rates even if there is a lesser impact on patients’ symptoms, functional status, and HRQoL [26]. This study therefore used both cut-off values of ≥ 2 and ≥ 3 concurrent diseases to define multimorbidity.

HRQoL was measured using the EQ-5D-5L, the most widely used preference-based instrument for measuring HRQoL [21] and recommended by Belgian guidelines for economic evaluations in health care [27]. The EQ-5D-5L includes a descriptive system consisting of five dimensions: mobility, self-care, usual activities, pain/discomfort, and anxiety/depression. Each dimension has five response categories (no problems, slight problems, moderate problems, severe problems, and extreme problems/unable to), from which a single index value or utility score can be calculated ranging between 0 (death) and 1 (perfect health). Negative values can also be generated for health states perceived worse than death. Converting the dimension scores into a single index value requires a country-specific algorithm based on population-level preferences for different health states. In Belgium, however, such an algorithm is so far only available for the EQ-5D-3L encompassing only three answering possibilities [28]. A cross-walk function was therefore used to map the EQ-5D-5L health states to EQ-5D-3L health states, resulting in index values ranging from − 0.158 to 1 [29, 30]. The EQ-VAS, measuring respondents’ self-rated health on a 0–100 scale, was not included in the BHIS 2018.

The following socio-demographic data were used: age (15 to 101 years), sex (male, female), educational attainment (no diploma, lower education, lower secondary education, higher secondary education, post-secondary education, higher education, doctoral degree), civil status (single, married or legally cohabiting, widow(er), divorced), and region (Brussels, Flanders, Wallonia). Socio-economic status was based on the highest level of education of the household and classified into low (lower secondary education or less), intermediate (higher secondary education), and high (higher education). Only participants who completed the EQ-5D-5L were included.

### Statistical analyses

Statistics were undertaken using IBM SPSS statistical software (version 26.0). The design effects of the survey were taken into account to deal with the complex design of the BHIS [23], resulting in representativeness for the total population, at national, regional, and provincial level.

A contingency table in matrix form was constructed to determine patterns of combinations of diseases. To increase robustness, only combinations occurring ≥ 2.5% (arbitrary cut-off) in the population were considered here.

We fitted linear mixed models to analyze two-way and three-way interaction effects (i.e. effect modifications) of disease combinations on HRQoL. The EQ-5D-5L index score was the dependent variable in all models. The individual diseases were included as fixed effects. Each model also contained a two-way or three-way interaction term plus all underlying interactions for the dyad and triad combinations, respectively. The following design effects of the survey were taken into account: clustering at household level as random effect (random intercept for household), regional stratification as fixed factor, and survey weights as weighting. Normality of the residuals was assessed by Q-Q plot. We relied on the central limit theorem, given our large sample size. Significance of the interaction between two comorbidities indicates that the effect of one comorbidity is different in the presence or absence of the other comorbidity. A negative interaction (i.e. synergistic effect) shows that the combined effect of two comorbidities was associated with an estimated mean decrease in HRQoL that is higher (stronger/more pronounced) than expected based on the additive effect of the comorbidities separately. Conversely, a positive interaction (i.e. antagonistic effect) indicates that the combined effect of two comorbidities was associated with an estimated mean decrease in HRQoL that is less than expected based on the additive effect of each of them individually. It is important to highlight that effect modification is bidirectional meaning that each disease simultaneously influences, and is influenced by, the other disease. Estimated marginal mean HRQoL scores for each disease combination were calculated from the final model. The regression models were not adjusted for several covariates (e.g. age, sex, educational attainment), because the aim was not to find the model with the most ideal goodness-of-fit, but rather to depict the actual situation. P-values have not been adjusted for multiplicity. Statistical significance was set at *P* < 0.05, trends towards significance (*P* < 0.1) were also presented.

## Results

### Sample characteristics

The EQ-5D-5L was completed by 85% of participants (n = 7,509). Descriptive statistics are outlined in Table 2. The mean age was 48.6 years, and slightly more than half were women (51.6%). About half of the participants had a high socioeconomic status (50.8%), whilst 16.8% had a low socioeconomic status. Approximately 30% had no chronic disease and 23.4% had one chronic disease. Multimorbidity occurred in 46.7% (≥ 2 conditions) and 29.7% (≥ 3 conditions) of the participants. Multimorbidity was more prevalent in women and increased with age and lower socioeconomic status. Females, older persons, and persons with lower socioeconomic status had lower HRQoL. Moreover, HRQoL decreased considerably with the presence of multiple chronic diseases. Participants with ≥ 3 chronic diseases (0.65) reported a considerably lower HRQoL score compared to participants with two chronic diseases (0.79) or with one chronic disease (0.85). Participants without chronic disease reported the highest HRQoL score (0.90).

**Table 2:** Characteristics of the study participants (N = 7,509) by chronic disease status, survey-weighted

	Overall		0 chronic disease		1 chronic disease		2 chronic diseases		≥ 3 chronic diseases	
	%	EQ-5D-5L (SD)	%	EQ-5D-5L (SD)	%	EQ-5D-5L (SD)	%	EQ-5D-5L (SD)	%	EQ-5D-5L (SD)
			30.0%	0.90 (0.14)	23.4%	0.85 (0.16)	17.0%	0.79 (0.18)	29.7%	0.65 (0.22)
Age, mean (SD)	48.6 (18.88)		40.0 (16.91)		45.3 (17.33)		51.0 (17.76)		58.5 (17.65)	
15–24 years	11.7%	0.85 (0.18)	20.4%	0.89 (0.15)	13.0%	0.88 (0.15)	8.4%	0.76 (0.25)	3.7%	0.72 (0.18)
25–44 years	31.6%	0.83 (0.18)	42.4%	0.91 (0.13)	36.9%	0.84 (0.16)	29.4%	0.79 (0.16)	17.6%	0.66 (0.20)
45–64 years	35.1%	0.78 (0.20)	28.3%	0.88 (0.14)	35.9%	0.85 (0.16)	38.7%	0.78 (0.17)	39.4%	0.65 (0.22)
≥ 65 years	21.6%	0.72 (0.24)	8.9%	0.86 (0.16)	14.1%	0.84 (0.18)	23.5%	0.79 (0.18)	39.3%	0.63 (0.24)
Sex										
Female	51.6%	0.77 (0.21)	46.4%	0.88 (0.15)	46.7%	0.83 (0.17)	51.3%	0.77 (0.17)	60.9%	0.64 (0.22)
Male	48.4%	0.82 (0.20)	53.6%	0.91 (0.13)	53.3%	0.87 (0.16)	48.7%	0.80 (0.19)	39.1%	0.66 (0.23)
Socioeconomic status										
Low	16.8%	0.71 (0.26)	12.0%	0.88 (0.16)	14.1%	0.83 (0.19)	16.8%	0.72 (0.19)	23.6%	0.57 (0.27)
Intermediate	32.4%	0.78 (0.21)	31.5%	0.89 (0.15)	30.2%	0.83 (0.17)	33.2%	0.79 (0.20)	34.6%	0.64 (0.22)
High	50.8%	0.83 (0.17)	56.6%	0.90 (0.13)	55.6%	0.87 (0.15)	49.9%	0.81 (0.16)	41.8%	0.70 (0.19)
Civil status										
Single	29.3%	0.82 (0.20)	40.2%	0.90 (0.15)	32.0%	0.85 (0.16)	24.3%	0.77 (0.21)	19.0%	0.64 (0.22)
Married or legally cohabiting	54.3%	0.80 (0.19)	50.2%	0.90 (0.13)	54.9%	0.86 (0.15)	58.4%	0.80 (0.16)	55.8%	0.68 (0.21)
Widow(er)	6.7%	0.67 (0.27)	3.6%	0.86 (0.18)	4.4%	0.83 (0.20)	6.2%	0.73 (0.18)	12.0%	0.55 (0.28)
Divorced	9.7%	0.74 (0.23)	6.1%	0.88 (0.16)	8.7%	0.82 (0.19)	11.1%	0.78 (0.18)	13.2%	0.63 (0.23)
Region										
Flanders	58.6%	0.82 (0.19)	54.2%	0.92 (0.12)	64.0%	0.87 (0.15)	60.8%	0.82 (0.17)	57.4%	0.68 (0.21)
Brussels	9.0%	0.79 (0.21)	11.3%	0.88 (0.15)	8.6%	0.83 (0.18)	7.8%	0.76 (0.17)	7.8%	0.63 (0.24)
Wallonia	32.4%	0.75 (0.22)	34.5%	0.86 (0.16)	27.4%	0.81 (0.18)	31.4%	0.73 (0.18)	34.8%	0.60 (0.23)

### Single chronic diseases

The three most common chronic diseases were dorsopathies (31.4%), hypertension/high cholesterol (28.6%), and arthropathies (22%) (Table 3). On the other hand, stroke and hip fracture had a prevalence lower than 1%. HRQoL scores varied between diseases. The highest HRQoL score was reported in persons with allergy (0.75), followed by hypertension/high cholesterol (0.73), and thyroid problems (0.71). The lowest HRQoL score was reported in persons with depression (0.53), followed by stroke (0.54), and chronic fatigue (0.58).

**Table 3:** Prevalence and estimated mean health-related quality of life (HRQoL) score for single chronic diseases

	Total (%)	Women (%)	Mean age	Est. mean HRQoL score
Hip fracture	0.5	58.3	70.7	0.61 (0.54–0.68)
Stroke	0.6	27.4	69.6	0.54 (0.48–0.60)
Cirrhosis of the liver	1.0	62.8	60.1	0.64 (0.60–0.69)
Gallstones	1.0	84.6	54.2	0.69 (0.64–0.73)
Kidney disease	1.0	68.0	55.6	0.70 (0.66–0.74)
Cancer	2.2	60.1	61.5	0.65 (0.62–0.68)
Osteoporosis	3.3	89.2	69.0	0.61 (0.59–0.64)
Stomach ulcer	3.4	64.7	51.4	0.66 (0.63–0.68)
Chronic skin disease	4.0	58.5	49.4	0.68 (0.65–0.70)
Eye disease	4.4	57.2	70.9	0.67 (0.65–0.69)
Bowel disorder	4.7	68.8	55.2	0.63 (0.61–0.66)
Cardiovascular disease	5.8	46.8	66.8	0.64 (0.62–0.66)
Diabetes	6.0	48.2	64.7	0.67 (0.65–0.69)
Thyroid problems	7.0	84.2	56.7	0.71 (0.69–0.73)
Depression	7.4	63.3	50.2	0.53 (0.52–0.55)
Chronic fatigue	8.3	63.5	49.7	0.58 (0.57–0.60)
Respiratory disease	8.4	58.1	52.3	0.68 (0.66–0.69)
Neurological disorder	11.5	65.4	44.6	0.69 (0.68–0.71)
Genitourinary problems	15.7	52.2	63.4	0.64 (0.62–0.65)
Allergy	20.0	56.4	45.6	0.75 (0.74–0.76)
Arthropathies	22.0	60.7	62.2	0.65 (0.64–0.66)
Hypertension/high cholesterol	28.6	51.6	61.2	0.73 (0.72–0.73)
Dorsopathies	31.4	56.0	53.5	0.67 (0.67–0.68)

### Dyads of chronic diseases

**Fig. 1:**
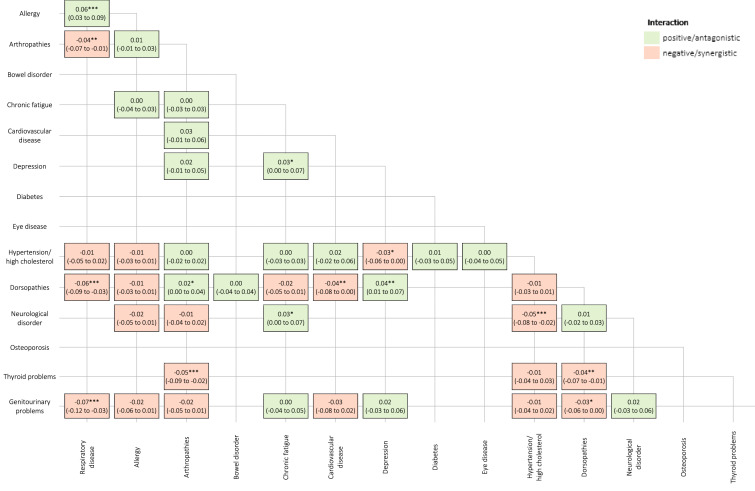
Interaction effects of the 41 chronic disease dyad combinations on health-related quality of life. *P-value < 0.1; **P-value < 0.05; ***P-value < 0.01

**Fig. 2:**
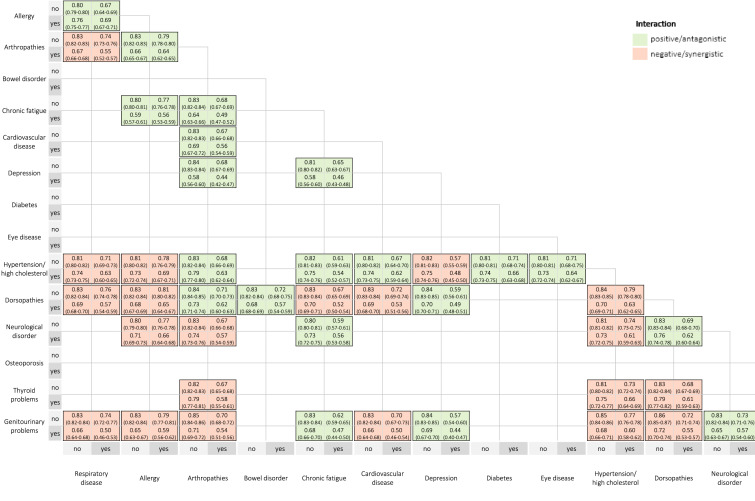
Estimated mean health-related quality of life scores for the 41 chronic disease dyad combinations

The prevalence matrix generated 41 dyad combinations with a prevalence ≥ 2.5% (Online Appendix 1). The most prevalent dyads were arthropathies + dorsopathies (14.6%), hypertension/high cholesterol + dorsopathies (12.8%), and hypertension/high cholesterol + arthropathies (11.8%). Figure 1 and Fig. 2 summarize the interaction effects and estimated mean HRQoL scores for the 41 dyads respectively.

The highest HRQoL score was reported in persons with allergy + respiratory disease (0.69) and hypertension/high cholesterol + allergy (0.69). The lowest HRQoL score was reported in persons with depression + arthropathies (0.44) and depression + genitourinary problems (0.44).

Based on our data, effect modification was observed for a minority of combinations (14 out of 41 pairs) and showed a mixed pattern: some interactions were negative/synergistic, some interactions were positive/antagonistic. The condition appearing the most frequently in significant disease pair interactions was dorsopathies, followed by respiratory disease and arthropathies.

The majority of disease pairs (9 out of 14 pairs) showed negative/synergistic interactions. The strongest negative/synergistic associations were found for dorsopathies + respiratory disease (*P* < 0.01), hypertension/high cholesterol + neurological disorder (*P* < 0.01), arthropathies + thyroid problems (*P* < 0.01), and genitourinary problems + respiratory disease (*P* < 0.01). The estimated mean decrease in HRQoL is also stronger in the presence of the following coexisting conditions: arthropathies + respiratory disease (*P* < 0.05), dorsopathies + cardiovascular disorder (*P* < 0.05), dorsopathies + thyroid problems (*P* < 0.05), hypertension/high cholesterol + depression (*P* < 0.1), and dorsopathies + genitourinary problems (*P* < 0.1).

The strongest positive/antagonistic association was observed for respiratory disease + allergy (*P* < 0.01). The estimated mean decrease in HRQoL is also smaller in the presence of the following coexisting conditions: depression + dorsopathies (*P* < 0.05), arthropathies + dorsopathies (*P* < 0.1), chronic fatigue + neurological disorder (*P* < 0.1), depression + chronic fatigue (*P* < 0.1).

### Triads of chronic diseases

**Fig. 3:**
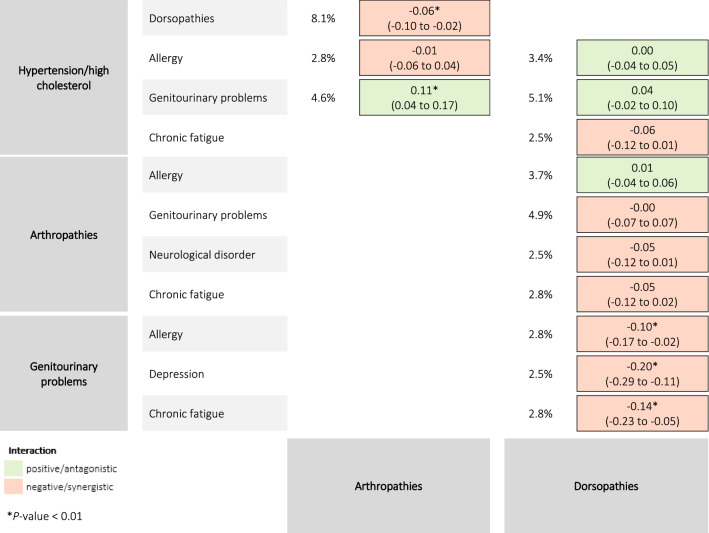
Prevalence and interaction effects of the 13 chronic disease triad combinations on health-related quality of life

**Fig. 4:**
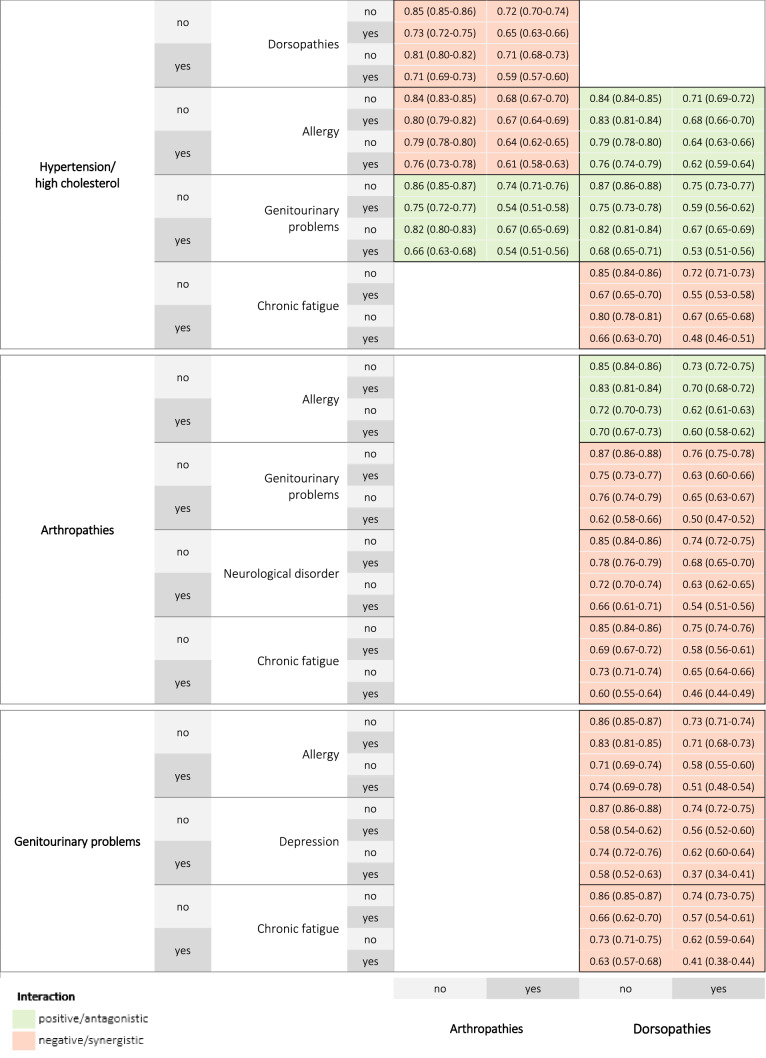
Estimated mean health-related quality of life scores for the 13 chronic disease triad combinations

Thirteen triad combinations with a prevalence ≥ 2.5% were observed. The most frequent triad combinations were hypertension/high cholesterol + arthropathies + dorsopathies (8.1%), hypertension/high cholesterol + genitourinary problems + dorsopathies (5.1%), and dorsopathies + arthropathies + genitourinary problems (4.9%). Figure 3 and Fig. 4 summarize the interaction effects and estimated mean HRQoL scores for the thirteen triads respectively.

The highest HRQoL score was reported in persons with allergy + hypertension/high cholesterol + dorsopathies (0.62). The lowest HRQoL score was reported in persons with depression + genitourinary problems + dorsopathies (0.37).

Five triad combinations showed significant interactions (*P* < 0.01). Negative/synergistic interactions were found for genitourinary problems + chronic fatigue + dorsopathies, genitourinary problems + depression + dorsopathies, genitourinary problems + allergy + dorsopathies, and hypertension/high cholesterol + arthropathies + dorsopathies. A positive/antagonistic interaction was found for hypertension/high cholesterol + genitourinary problems + arthropathies.

## Discussion

This study estimated the impact of multimorbidity patterns on HRQoL in a representative sample of the Belgian population. Interaction effects of coexisting conditions were estimated based on self-reported data across 23 chronic diseases or disease groups. With these results, we aimed at meeting the rising demand from epidemiologists, clinicians, and policy makers for a better understanding of the burden of chronic diseases, in particular the clinical and societal impact of multimorbidity. In addition, health economists can use the results in their cost-effectiveness analysis supporting reimbursement decisions. The joint effects of disease combinations on HRQoL have been explored in several studies, however, interaction estimates for specific conditions with low prevalence rates measured by the EQ-5D-5L have not been previously investigated in such detail [14, 31–33]. Moreover, to the best of our knowledge, this study is the first to investigate interaction effects of triad disease combinations on HRQoL measured by the EQ-5D-5L.

Our results show that the estimated mean HRQoL is lower when a chronic disease is present compared to when it is absent. Chronically ill patients had lower HRQoL scores, ranging from 0.53 to 0.75, compared to the reference value (0.84) from the Belgian general population in 2018 [34]. Indeed, a large body of evidence confirms the substantial reduction in HRQoL across chronic diseases [35].

Multimorbidity was highly prevalent, which may be attributed to the large number of chronic diseases included [36]. A review of prevalence studies identified estimates ranging between less than 5% to more than 95%, often due to discrepancies in the operational definition and cut-off value used to define multimorbidity, which makes comparison across populations difficult [26, 36]. More importantly, there is still no universally accepted definition for what constitutes to ‘chronic’ conditions in multimorbidity research. Hence, this study included all conditions assessed in the BHIS, even though some conditions are contentious (e.g. hip fracture, allergy) [37]. Moreover, the prevalence of multimorbidity is determined by the population studied (e.g. higher prevalence in older populations) and depends on the setting (e.g. higher prevalence in primary care than in the general population) [26, 36, 38]. Despite methodological variability, our multimorbidity pattern is more or less similar to other findings [17, 39, 40].

Disability-adjusted life year (DALY) is used as key metric to estimate overall disease burden [41]. At present, three comorbidity adjustment approaches exist within the burden of disease context, i.e. additive, multiplicative, and maximum approach [42]. However, these approaches ignore the probability that a combined effect can be higher than the sum of the independent condition effects [42]. Conversely, our results showed that synergistic effects were more common within multimorbidity patterns. Besides, we demonstrated novel synergistic effects. More specifically, we identified nine pairs with negative interaction effects on HRQoL, of which four were sufficiently large and precisely estimated. These results confirm the evidence that the impact of chronic diseases on HRQoL is not only reflected by the simple sum of the diseases [31–33, 43]. A possible mechanism for explaining these synergistic effects is that treatment for one condition could adversely affect another pre-existing condition, resulting in a greater reduction in HRQoL [44]. Moreover, coexisting conditions may affect different body systems and HRQoL dimensions, for example low back problems and depression [32]. Results of previous studies on the joint effect of diseases are heterogeneous. Synergetic interactions of combinations such as diabetes + cardiovascular disease, stroke + coronary disease or diabetes + hypertension were not observed in this study [31, 32, 45].

Our results also identified significant antagonistic interactions. It is remarkable that antagonistic interactions were found in more subjective diseases such as depression and chronic fatigue. A possible explanation for subtractive effects can be related to the concept of ‘response shift’, which reflects patients’ adaptation to their new life circumstances as a result of changing health [46]. These adaptations may help to buffer the impact of a second medical condition, resulting in a smaller decrement in HRQoL than would otherwise be expected [47]. Another plausible explanation could be the healthy-responder effect, indicating that persons with, for example, more severe depression may be underrepresented, leading to higher HRQoL scores [47]. Moreover, several combinations (e.g. respiratory disease + allergy, chronic fatigue + depression) have similar symptoms and common aetiology, hence additional diagnosis has less impact on HRQoL [32]. However, the latter can also be attributed to ‘response bias’; people may have indicated one condition under several other denominators because of unclear diagnosis, which results in double reporting. A final explanation is that pain relief medicines or antidepressants for one condition may have beneficial effects on other coexisting conditions.

Literature suggest that the effects of multimorbidity on HRQoL may be more complex than observed by pairwise interaction terms [31]. Nevertheless, estimations of three-way interactions are largely understudied due to insufficient observations and the difficult interpretation [31]. This study considered three-way combinations to fully capture higher order interactions. As a result, significant interactions were found for five triad combinations. Analogous to the results of the dyad combinations, we observed a dominant presence of synergistic effects. These results suggest that any additional condition is commonly associated with greater reduction in HRQoL. To date, there are no studies that have investigated the interaction between triad combinations on HRQoL, hence our results could not be compared with previous findings.

### Sensitivity analysis

Recently, an EQ-5D-5L value set for Belgium has been developed [48]. As such, we performed a sensitivity analysis to assess the impact of the new value set on results and conclusions. The analysis showed that results systematically differed according to the value set used. The EQ-5D-5L value set produces higher values overall and across all conditions included. These results are not unexpected because similar findings were found in other countries (e.g. England, The Netherlands, Spain) with higher utility values for the EQ-5D-5L value set compared to the crosswalk value set [49–51]. In addition, the new value set generates 12 additional significant interaction effects for dyad disease combinations (Online Appendix 2). In detail, four significant positive/antagonistic interactions have disappeared, 16 significant negative/synergistic interactions have been added. Moreover, mainly disease combinations with depression and chronic fatigue changed from positive/antagonistic interactions to negative/synergistic interactions, with stronger significance levels. In general, only disease combinations that fall within the same disease classification system (i.e. allergy + respiratory disease, hypertension/high cholesterol + cardiovascular disease) have an antagonistic relationship. As such, we can conclude that there will always be an synergistic/negative HRQoL effect, making multimorbidity even more important than initially thought. Possible reasons for the observed differences may be attributed to changes in for example population demographics, preferences over time, descriptive system of the EQ-5D, and valuation method used [49, 51]. The magnitude of differences between value sets may however have important implications for decision-making as they can greatly impact estimates (e.g. QALYs) of health economic evaluations.

### Strengths and limitations

This study has several major strengths. A first strength is that the BHIS data is based on a large, representative sample of the general population which strengthens external validity. A second strength is the availability of a Belgian value set for the EQ-5D-3L because guidelines recommend country-specific value sets to estimate HRQoL more precisely within a country. Another strength is the large sample of morbidity across 38 long-term conditions, which allowed the assessment of interaction effects of coexisting conditions with low prevalence often not detectable in smaller studies [31, 52]. Nevertheless, grouping the 38 diseases into 23 disease groups may have masked potential interactions between diseases within larger disease groups, however, if synergistic or antagonistic effects were present, it is also expected to detect them across disease groups [17].

Some limitations should be considered. A first limitation is that the presence of chronic diseases was based on self-reports. Accurate self-reports require sufficient knowledge of participants to report on medical conditions. This is challenging because patients are often confused to distinguish between symptoms and the actual disease, and because some diseases are very subjective (e.g. chronic fatigue) [53]. In addition, it is possible that people indicate that they have several diseases because these conditions share homogeneous symptoms and common aetiology. Another important concern when using self-reports, especially over a 12-month time period, is recall bias [54]. Self-reports are not as valid and reliable as medical records because respondents may underestimate/overestimate the prevalence of medical conditions and risk behaviours [55–59]. However, several studies found a high concordance between self-reports and medical records when measuring the prevalence of multimorbidity [60–64]. As well, self-reported data are the most appropriate alternative in the absence of objective diagnoses. Another limitation is the non-exhaustive list of chronic diseases included in the BHIS. This implies missing other important diseases such as Alzheimer's disease, which has previously been found to greatly impact HRQoL [65, 66]. Besides, a minority of mental or psychiatric conditions were included in the list. Furthermore, despite our relatively large sample size, some diseases were underrepresented, which might produce bias and decreasing statistical power. Another limitation is that disease duration has not been taken into account. Studies found worse HRQoL in specific diseases with longer disease duration, whilst other studies showed that longer disease duration was associated with better HRQoL [67]. Another limitation is that the EQ-5D-5L covers relatively few dimensions of HRQoL that are disease-specific. The EQ-5D may therefore be insensitive to health problems (e.g. nausea, sleep disturbance) experienced by specific diseases [68]. Moreover, this study aimed for representativeness, hence the regression models were not corrected for several covariates (e.g. age, sex, educational attainment). A final limitation is the sampling procedure in the BHIS which can imply selection bias.

## Conclusions

This study revealed that diverse multimorbidity patterns were synergistically or antagonistically associated with lower HRQoL. Given that multimorbidity will emerge in the coming decades, tackling its burden is needed, especially because most disease combinations affect each other synergistically, resulting in a greater reduction in HRQoL. Further knowledge about those combinations with a greater impact on HRQoL as well as knowledge on the complex relationship between HRQoL and multimorbidity is needed to better understand disease burden beyond mortality and morbidity data and to guide health care providers in their clinical practice and policy makers in their priority setting regarding multiple disease management decisions.

## Supplementary Information

Below is the link to the electronic supplementary material.

Supplementary file1 (DOCX 55 kb)

Supplementary file2 (DOCX 37 kb)

## Abbreviations

BHIS: Belgian Health Interview Survey
DALY: Disability-adjusted life years
EQ-5D-5L: Five-level EuroQol five-dimensional questionnaire
HRQoL: Health-related quality of life
ICD-10: International Classification of Diseases, 10th revision
QALY: Quality-adjusted life year
