# Supplementary material for: The impact of multimorbidity patterns on health-related quality of life in the general population: results of the Belgian Health Interview Survey
Source: Qual Life Res. 2021 Aug 23;31(2):551–65. doi: 10.1007/s11136-021-02951-w (PMC8847309; doi:10.1007/s11136-021-02951-w)
Supplement: Supplementary file 1 — Supplementary file1 (DOCX 55 kb) [file 11136_2021_2951_MOESM1_ESM.docx]

| Cardiovascular disease | 1.3% |  |  |  |  |  |  |  |  |  |  |  |  |  |  |  |  |  |  |  |  |  |
| --- | --- | --- | --- | --- | --- | --- | --- | --- | --- | --- | --- | --- | --- | --- | --- | --- | --- | --- | --- | --- | --- | --- |
| Hypertension/high cholesterol | 3.6% | 3.8% |  |  |  |  |  |  |  |  |  |  |  |  |  |  |  |  |  |  | 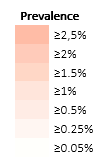 |  |
| Stroke | 0.2% | 0.3% | 0.4% |  |  |  |  |  |  |  |  |  |  |  |  |  |  |  |  |  |  |  |
| Arthropathies | 3.0% | 2.8% | 11.8% | 0.4% |  |  |  |  |  |  |  |  |  |  |  |  |  |  |  |  |  |  |
| Dorsopathies | 3.9% | 2.8% | 12.8% | 0.3% | 14.6% |  |  |  |  |  |  |  |  |  |  |  |  |  |  |  |  |  |
| Diabetes | 0.8% | 1.1% | 4.2% | 0.2% | 2.4% | 2.4% |  |  |  |  |  |  |  |  |  |  |  |  |  |  |  |  |
| Allergy | 3.8% | 1.4% | 5.8% | 0.2% | 5.1% | 8.5% | 1.2% |  |  |  |  |  |  |  |  |  |  |  |  |  |  |  |
| Stomach ulcer | 0.9% | 0.6% | 1.5% | 0.0% | 1.5% | 1.8% | 0.6% | 1.2% |  |  |  |  |  |  |  |  |  |  |  |  |  |  |
| Cirrhosis of the liver | 0.1% | 0.2% | 0.7% | 0.0% | 0.5% | 0.5% | 0.2% | 0.3% | 0.1% |  |  |  |  |  |  |  |  |  |  |  |  |  |
| Cancer | 0.3% | 0.4% | 0.9% | 0.1% | 0.7% | 1.1% | 0.2% | 0.7% | 0.2% | 0.1% |  |  |  |  |  |  |  |  |  |  |  |  |
| Neurological disorder | 1.6% | 1.0% | 3.6% | 0.1% | 3.1% | 5.8% | 0.8% | 3.8% | 1.0% | 0.3% | 0.6% |  |  |  |  |  |  |  |  |  |  |  |
| Genitourinary problems | 2.8% | 2.9% | 8.5% | 0.4% | 6.7% | 8.5% | 2.3% | 4.1% | 1.2% | 0.5% | 1.2% | 3.1% |  |  |  |  |  |  |  |  |  |  |
| Depression | 1.6% | 0.8% | 3.0% | 0.2% | 2.7% | 4.5% | 0.6% | 2.2% | 0.8% | 0.2% | 0.6% | 2.2% | 3.4% |  |  |  |  |  |  |  |  |  |
| Thyroid problems | 1.0% | 0.6% | 3.2% | 0.1% | 2.8% | 3.3% | 0.8% | 2.0% | 0.5% | 0.2% | 0.3% | 1.1% | 2.0% | 1.1% |  |  |  |  |  |  |  |  |
| Eye disease | 0.6% | 0.8% | 2.8% | 0.0% | 2.2% | 2.1% | 0.8% | 1.1% | 0.2% | 0.1% | 0.2% | 0.6% | 1.7% | 0.4% | 0.7% |  |  |  |  |  |  |  |
| Chronic fatigue | 1.7% | 1.0% | 3.4% | 0.2% | 3.4% | 5.3% | 0.9% | 2.7% | 1.1% | 0.3% | 0.7% | 2.9% | 3.7% | 3.0% | 1.3% | 0.5% |  |  |  |  |  |  |
| Osteoporosis | 0.5% | 0.5% | 1.8% | 0.1% | 2.3% | 2.1% | 0.5% | 0.8% | 0.3% | 0.1% | 0.2% | 0.4% | 1.2% | 0.6% | 0.6% | 0.5% | 0.6% |  |  |  |  |  |
| Hip fracture | 0.0% | 0.1% | 0.2% | 0.0% | 0.3% | 0.2% | 0.0% | 0.1% | 0.0% | 0.0% | 0.0% | 0.1% | 0.1% | 0.1% | 0.1% | 0.1% | 0.0% | 0.1% |  |  |  |  |
| Bowel disorder | 1.0% | 0.7% | 2.1% | 0.2% | 2.2% | 2.9% | 0.5% | 1.6% | 0.8% | 0.3% | 0.5% | 1.1% | 2.4% | 1.1% | 0.7% | 0.4% | 1.8% | 05% | 0.1% |  |  |  |
| Kidney disease | 0.2% | 0.2% | 0.5% | 0.0% | 0.4% | 0.5% | 0.1% | 0.3% | 0.1% | 0.1% | 0.1% | 0.3% | 0.6% | 0.2% | 0.2% | 0.1% | 0.2% | 0.1% | 0.0% | 0.2% |  |  |
| Chronic skin disease | 0.7% | 0.4% | 1.5% | 0.0% | 1.4% | 1.9% | 0.4% | 2.0% | 0.5% | 0.1% | 0.4% | 0.9% | 1.5% | 0.7% | 0.5% | 0.3% | 0.9% | 0.3% | 0.0% | 0.5% | 0.0% |  |
| Gall stones | 0.2% | 0.1% | 0.5% | 0.0% | 0.4% | 0.5% | 0.1% | 0.4% | 0.1% | 0.1% | 0.0% | 0.1% | 0.4% | 0.1% | 0.1% | 0.1% | 0.2% | 0.0% | 0.0% | 0.1% | 0.0% | 0.1% |
|  | Respiratory disease | Cardiovascular disease | Hypertension/high cholesterol | Stroke | Arthropathies | Dorsopathies | Diabetes | Allergy | Stomach ulcer | Cirrhosis of the liver | Cancer | Neurological disorder | Genitourinary problems | Depression | Thyroid problems | Eye disease | Chronic fatigue | Osteoporosis | Hip fracture | Bowel disorder | Kidney disease | Chronic skin disease |

**Appendix 1. Prevalence matrix of the chronic disease dyad combinations (relative frequency distribution).**
