# Supplementary material for: The impact of multimorbidity patterns on health-related quality of life in the general population: results of the Belgian Health Interview Survey
Source: Qual Life Res. 2021 Aug 23;31(2):551–65. doi: 10.1007/s11136-021-02951-w (PMC8847309; doi:10.1007/s11136-021-02951-w)
Supplement: Supplementary file 2 — Supplementary file2 (DOCX 37 kb) [file 11136_2021_2951_MOESM2_ESM.docx]

| **Table 1. Characteristics of the study participants (N = 7,509) by chronic disease status, survey-weighted. New EQ-5D-5L value set.** | | | | | | | | | | |
| --- | --- | --- | --- | --- | --- | --- | --- | --- | --- | --- |
|  | **Overall** | | **0 chronic disease** | | **1 chronic disease** | | **2 chronic diseases** | | **≥ 3 chronic diseases** | |
|  | % | EQ-5D-5L (SD) | % | EQ-5D-5L (SD) | % | EQ-5D-5L (SD) | % | EQ-5D-5L (SD) | % | EQ-5D-5L (SD) |
|  |  |  | 30.0% | 0.93 (0.11) | 23.4% | 0.90 (0.14) | 17.0% | 0.85 (0.17) | 29.7% | 0.70 (0.28) |
| **Age, mean (SD)** | 48.6 (18.88) |  | 40.0 (16.91) |  | 45.3 (17.33) |  | 51.0 (17.76) |  | 58.5 (17.65) |  |
| 15-24 years | 11.7% | 0.90 (0.16) | 20.4% | 0.93 (0.12) | 13.0% | 0.92 (0.13) | 8.4% | 0.82 (0.27) | 3.7% | 0.79 (0.20) |
| 25-44 years | 31.6% | 0.88 (0.17) | 42.4% | 0.95 (0.09) | 36.9% | 0.90 (0.15) | 29.4% | 0.86 (0.15) | 17.6% | 0.72 (0.25) |
| 45-64 years | 35.1% | 0.83 (0.22) | 28.3% | 0.93 (0.12) | 35.9% | 0.90 (0.13) | 38.7% | 0.84 (0.16) | 39.4% | 0.71 (0.28) |
| ≥ 65 years | 21.6% | 0.77 (0.27) | 8.9% | 0.91 (0.12) | 14.1% | 0.89 (0.16) | 23.5% | 0.86 (0.16) | 39.3% | 0.68 (0.30) |
| **Sex** |  |  |  |  |  |  |  |  |  |  |
| Female | 51.6% | 0.87 (0.20) | 46.4% | 0.92 (0.12) | 46.7% | 0.89 (0.15) | 51.3% | 0.85 (0.16) | 60.9% | 0.69 (0.28) |
| Male | 48.4% | 0.82 (0.22) | 53.6% | 0.94 (0.10) | 53.3% | 0.91 (0.14) | 48.7% | 0.86 (0.19) | 39.1% | 0.72 (0.28) |
| **Socioeconomic status** |  |  |  |  |  |  |  |  |  |  |
| Low | 16.8% | 0.75 (0.30) | 12.0% | 0.92 (0.15) | 14.1% | 0.88 (0.18) | 16.8% | 0.79 (0.21) | 23.6% | 0.60 (0.35) |
| Intermediate | 32.4% | 0.83 (0.22) | 31.5% | 0.93 (0.12) | 30.2% | 0.88 (0.16) | 33.2% | 0.85 (0.19) | 34.6% | 0.69 (0.27) |
| High | 50.8% | 0.88 (0.16) | 56.6% | 0.94 (0.09) | 55.6% | 0.92 (0.12) | 49.9% | 0.87 (0.13) | 41.8% | 0.77 (0.22) |
| **Civil status** |  |  |  |  |  |  |  |  |  |  |
| Single | 29.3% | 0.86 (0.20) | 40.2% | 0.93 (0.11) | 32.0% | 0.90 (0.14) | 24.3% | 0.83 (0.23) | 19.0% | 0.69 (0.28) |
| Married or legally cohabiting | 54.3% | 0.86 (0.19) | 50.2% | 0.94 (0.09) | 54.9% | 0.91 (0.13) | 58.4% | 0.86 (0.14) | 55.8% | 0.74 (0.25) |
| Widow(er) | 6.7% | 0.72 (0.32) | 3.6% | 0.90 (0.15) | 4.4% | 0.88 (0.19) | 6.2% | 0.81 (0.18) | 12.0% | 0.59 (0.36) |
| Divorced | 9.7% | 0.79 (0.26) | 6.1% | 0.91 (0.17) | 8.7% | 0.87 (0.19) | 11.1% | 0.84 (0.17) | 13.2% | 0.66 (0.31) |
| **Region** |  |  |  |  |  |  |  |  |  |  |
| Flanders | 58.6% | 0.87 (0.19) | 54.2% | 0.95 (0.08) | 64.0% | 0.92 (0.12) | 60.8% | 0.88 (0.16) | 57.4% | 0.74 (0.26) |
| Brussels | 9.0% | 0.84 (0.23) | 11.3% | 0.93 (0.12) | 8.6% | 0.89 (0.17) | 7.8% | 0.84 (0.16) | 7.8% | 0.67 (0.31) |
| Wallonia | 32.4% | 0.80 (0.24) | 34.5% | 0.91 (0.13) | 27.4% | 0.86 (0.18) | 31.4% | 0.80 (0.18) | 34.8% | 0.64 (0.30) |

| **Table 2. Prevalence and estimated mean HRQoL score for single chronic diseases, based on EQ-5D crosswalk value set and the new EQ-5D-5L value set.** | | | |
| --- | --- | --- | --- |
|  |  | **Estimated mean HRQoL score** | |
|  | **Total %** | **EQ-5D crosswalk value set** | **New EQ-5D-5L value set** |
| Hip fracture | 0.5% | 0.61 | 0.64 |
| Stroke | 0.6% | 0.54 | 0.58 |
| Cirrhosis of the liver | 1.0% | 0.64 | 0.67 |
| Gallstones | 1.0% | 0.69 | 0.73 |
| Kidney disease | 1.0% | 0.70 | 0.73 |
| Cancer | 2.2% | 0.65 | 0.70 |
| Osteoporosis | 3.3% | 0.61 | 0.64 |
| Stomach ulcer | 3.4% | 0.66 | 0.70 |
| Chronic skin disease | 4.0% | 0.68 | 0.73 |
| Eye disease | 4.4% | 0.67 | 0.73 |
| Bowel disorder | 4.7% | 0.63 | 0.67 |
| Cardiovascular disease | 5.8% | 0.64 | 0.69 |
| Diabetes | 6.0% | 0.67 | 0.72 |
| Thyroid problems | 7.0% | 0.71 | 0.76 |
| Depression | 7.4% | 0.53 | 0.56 |
| Chronic fatigue | 8.3% | 0.58 | 0.61 |
| Respiratory disease | 8.4% | 0.68 | 0.72 |
| Neurological disorder | 11.5% | 0.69 | 0.75 |
| Genitourinary problems | 15.7% | 0.64 | 0.68 |
| Allergy | 20.0% | 0.75 | 0.80 |
| Arthropathies | 22.0% | 0.65 | 0.71 |
| Hypertension/high cholesterol | 28.6% | 0.73 | 0.78 |
| Dorsopathies | 31.4% | 0.67 | 0.73 |

| Allergy |  | 0.06*** | |  |  |  |  |  | |  |  | |  |  | |  |  | |  |  | |  |  | |  |  | |  |  | |  | 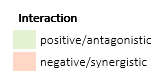 | |  |  | |  |  | |  |  | |
| --- | --- | --- | --- | --- | --- | --- | --- | --- | --- | --- | --- | --- | --- | --- | --- | --- | --- | --- | --- | --- | --- | --- | --- | --- | --- | --- | --- | --- | --- | --- | --- | --- | --- | --- | --- | --- | --- | --- | --- | --- | --- | --- |
|  |  | 0.06*** | |  |  |  |  |  |  |  |  |  |  |  |  |  |  |  |  |  |  |  |  |  |  |  |  |  |  |  |  |  |  |  |  |  |  |  |  |  |  |  |
|  |  |  |  |  |  |  |  |  | |  |  | |  |  | |  |  | |  |  | |  |  | |  |  | |  |  | |  |  | |  |  | |  |  | |  |  | |
| Arthropathies |  | -0.04** | |  | 0.01 | |  |  |  |  |  | |  |  | |  |  | |  |  | |  |  | |  |  | |  |  | |  |  | |  |  | |  |  | |  |  | |
|  |  | -0.07*** | |  | -0.00 | |  |  |  |  |  |  |  |  |  |  |  |  |  |  |  |  |  |  |  |  |  |  |  |  |  |  |  |  |  |  |  |  |  |  |  |  |
|  |  |  |  |  |  |  |  |  |  |  |  | |  |  | |  |  | |  |  | |  |  | |  |  | |  |  | |  |  | |  |  | |  |  | |  |  | |
| Bowel disorder |  |  |  |  |  |  |  |  |  |  |  |  |  |  | |  |  | |  |  | |  |  | |  |  | |  |  | |  |  | |  |  | |  |  | |  |  | |
|  |  |  |  |  |  |  |  |  |  |  |  |  |  |  |  |  |  |  |  |  |  |  |  |  |  |  |  |  |  |  |  |  |  |  |  |  |  |  |  |  |  |  |
|  |  |  |  |  |  |  |  |  |  |  |  |  |  |  | |  |  | |  |  | |  |  | |  |  | |  |  | |  |  | |  |  | |  |  | |  |  | |
| Chronic fatigue |  |  |  |  | 0.00 | |  | 0.00 | |  |  |  |  |  |  |  |  | |  |  | |  |  | |  |  | |  |  | |  |  | |  |  | |  |  | |  |  | |
|  |  |  |  |  | -0.01 | |  | -0.07*** | |  |  |  |  |  |  |  |  |  |  |  |  |  |  |  |  |  |  |  |  |  |  |  |  |  |  |  |  |  |  |  |  |  |
|  |  |  |  |  |  |  |  |  |  |  |  |  |  |  |  |  |  | |  |  | |  |  | |  |  | |  |  | |  |  | |  |  | |  |  | |  |  | |
| Cardiovascular disease |  |  |  |  |  |  |  | 0.03 | |  |  |  |  |  |  |  |  |  |  |  | |  |  | |  |  | |  |  | |  |  | |  |  | |  |  | |  |  | |
|  |  |  |  |  |  |  |  | 0.02 | |  |  |  |  |  |  |  |  |  |  |  |  |  |  |  |  |  |  |  |  |  |  |  |  |  |  |  |  |  |  |  |  |  |
|  |  |  |  |  |  |  |  |  |  |  |  |  |  |  |  |  |  |  |  |  | |  |  | |  |  | |  |  | |  |  | |  |  | |  |  | |  |  | |
| Depression |  |  |  |  |  |  |  | 0.02 | |  |  |  |  | 0.03* | |  |  |  |  |  |  |  |  | |  |  | |  |  | |  |  | |  |  | |  |  | |  |  | |
|  |  |  |  |  |  |  |  | -0.05** | |  |  |  |  | -0.04** | |  |  |  |  |  |  |  |  |  |  |  |  |  |  |  |  |  |  |  |  |  |  |  |  |  |  |  |
|  |  |  |  |  |  |  |  |  |  |  |  |  |  |  |  |  |  |  |  |  |  |  |  | |  |  | |  |  | |  |  | |  |  | |  |  | |  |  | |
| Diabetes |  |  |  |  |  |  |  |  |  |  |  |  |  |  |  |  |  |  |  |  |  |  |  |  |  |  | |  |  | |  |  | |  |  | |  |  | |  |  | |
|  |  |  |  |  |  |  |  |  |  |  |  |  |  |  |  |  |  |  |  |  |  |  |  |  |  |  |  |  |  |  |  |  |  |  |  |  |  |  |  |  |  |  |
|  |  |  |  |  |  |  |  |  |  |  |  |  |  |  |  |  |  |  |  |  |  |  |  |  |  |  | |  |  | |  |  | |  |  | |  |  | |  |  | |
| Eye disease |  |  |  |  |  |  |  |  |  |  |  |  |  |  |  |  |  |  |  |  |  |  |  |  |  |  |  |  |  | |  |  | |  |  | |  |  | |  |  | |
|  |  |  |  |  |  |  |  |  |  |  |  |  |  |  |  |  |  |  |  |  |  |  |  |  |  |  |  |  |  |  |  |  |  |  |  |  |  |  |  |  |  |  |
|  |  |  |  |  |  |  |  |  |  |  |  |  |  |  |  |  |  |  |  |  |  |  |  |  |  |  |  |  |  | |  |  | |  |  | |  |  | |  |  | |
| Hypertension/  high cholesterol |  | -0.01 | |  | -0.01 | |  | 0.00 | |  |  |  |  | 0.00 | |  | 0.02 | |  | -0.03* | |  | 0.01 | |  | 0.00 | |  |  |  |  |  | |  |  | |  |  | |  |  | |
|  |  | -0.04** | |  | -0.03** | |  | -0.01 | |  |  |  |  | -0.03** | |  | 0.006 | |  | -0.07*** | |  | -0.01 | |  | 0.01 | |  |  |  |  |  |  |  |  |  |  |  |  |  |  |  |
|  |  |  |  |  |  |  |  |  |  |  |  |  |  |  |  |  |  |  |  |  |  |  |  |  |  |  |  |  |  |  |  |  | |  |  | |  |  | |  |  | |
| Dorsopathies |  | -0.06*** | |  | -0.01 | |  | 0.02* | |  | 0.00 | |  | -0.02 | |  | -0.04** | |  | 0.04** | |  |  |  |  |  |  |  | -0.01 | |  |  |  |  |  | |  |  | |  |  | |
|  |  | -0.08*** | |  | -0.02 | |  | -0.01 | |  | -0.05** | |  | -0.07*** | |  | -0.07*** | |  | -0.03* | |  |  |  |  |  |  |  | -0.02** | |  |  |  |  |  |  |  |  |  |  |  |  |
|  |  |  |  |  |  |  |  |  |  |  |  |  |  |  |  |  |  |  |  |  |  |  |  |  |  |  |  |  |  |  |  |  |  |  |  | |  |  | |  |  | |
| Neurological disorder |  |  |  |  | -0.02 | |  | -0.01 | |  |  |  |  | 0.03* | |  |  |  |  |  |  |  |  |  |  |  |  |  | -0.05*** | |  | 0.01 | |  |  |  |  |  | |  |  | |
|  |  |  |  |  | -0.02 | |  | -0.05*** | |  |  |  |  | 0.004 | |  |  |  |  |  |  |  |  |  |  |  |  |  | -0.08*** | |  | -0.01 | |  |  |  |  |  |  |  |  |  |
|  |  |  |  |  |  |  |  |  |  |  |  |  |  |  |  |  |  |  |  |  |  |  |  |  |  |  |  |  |  |  |  |  |  |  |  |  |  |  | |  |  | |
| Osteoporosis |  |  |  |  |  |  |  |  |  |  |  |  |  |  |  |  |  |  |  |  |  |  |  |  |  |  |  |  |  |  |  |  |  |  |  |  |  |  |  |  |  | |
|  |  |  |  |  |  |  |  |  |  |  |  |  |  |  |  |  |  |  |  |  |  |  |  |  |  |  |  |  |  |  |  |  |  |  |  |  |  |  |  |  |  |  |
|  |  |  |  |  |  |  |  |  |  |  |  |  |  |  |  |  |  |  |  |  |  |  |  |  |  |  |  |  |  |  |  |  |  |  |  |  |  |  |  |  |  | |
| Thyroid problems |  |  |  |  |  |  |  | -0.05*** | |  |  |  |  |  |  |  |  |  |  |  |  |  |  |  |  |  |  |  | -0.01 | |  | -0.04** | |  |  |  |  |  |  |  |  |  |
|  |  |  |  |  |  |  |  | -0.09*** | |  |  |  |  |  |  |  |  |  |  |  |  |  |  |  |  |  |  |  | -0.02 | |  | -0.05** | |  |  |  |  |  |  |  |  |  |
|  |  |  |  |  |  |  |  |  |  |  |  |  |  |  |  |  |  |  |  |  |  |  |  |  |  |  |  |  |  |  |  |  |  |  |  |  |  |  |  |  |  |  |
| Genitourinary problems |  | -0.07*** | |  | -0.02 | |  | -0.02 | |  |  |  |  | 0.00 | |  | -0.03 | |  | 0.02 | |  |  |  |  |  |  |  | -0.01 | |  | -0.03* | |  | 0.02 | |  |  |  |  |  |  |
|  |  | -0.11*** | |  | -0.02 | |  | -0.07*** | |  |  |  |  | -0.07** | |  | -0.09*** | |  | -0.07** | |  |  |  |  |  |  |  | -0.04** | |  | -0.08*** | |  | -0.02 | |  |  |  |  |  |  |
|  |  |  |  |  |  |  |  |  |  |  |  |  |  |  |  |  |  |  |  |  |  |  |  |  |  |  |  |  |  |  |  |  |  |  |  |  |  |  |  |  |  |  |
|  |  | Respiratory disease | |  | Allergy | |  | Arthropathies | |  | Bowel disorder | |  | Chronic fatigue | |  | Cardiovascular disease | |  | Depression | |  | Diabetes | |  | Eye disease | |  | Hypertension/  high cholesterol | |  | Dorsopathies | |  | Neurological disorder | |  | Osteoporosis | |  | Thyroid problems | |

**Figure 1. Interaction effects of the 41 dyad combinations on HRQoL. Upper interaction: EQ-5D crosswalk value set; lower interaction: new EQ-5D-5L value set.** *P-value < 0.1; **P-value < 0.05; ***P-value < 0.01
